# Chemical Profile, Antioxidant, Anti-Inflammatory, and Anti-Cancer Effects of Italian *Salvia rosmarinus* Spenn. Methanol Leaves Extracts

**DOI:** 10.3390/antiox9090826

**Published:** 2020-09-03

**Authors:** Matteo Brindisi, Chouaha Bouzidi, Luca Frattaruolo, Monica R. Loizzo, Rosa Tundis, Annabelle Dugay, Brigitte Deguin, Anna Rita Cappello, Maria Stella Cappello

**Affiliations:** 1 Department of Pharmacy, Health and Nutritional Sciences, University of Calabria, Via Pietro Bucci, 87036 Rende (CS), Italy; matteo.brindisi@unical.it (M.B.); luca.frattaruolo@unical.it (L.F.); monica_rosa.loizzo@unical.it (M.R.L.); annarita.cappello@unical.it (A.R.C.); 2 Faculté de Pharmacie de Paris, Université de Paris, U.M.R. n 8038, —CiTCoM (CNRS, Université de Paris), 75006 Paris, France; chouaha.bouzidi@parisdescartes.fr (C.B.); annabelle.dugay@parisdescartes.fr (A.D.); brigitte.deguin@parisdescartes.fr (B.D.); 3 National Research Council (CNR), Institute of Science of Food Production (ISPA), Prov. le Lecce-Monteroni, 73100 Lecce, Italy; maristella.cappello@ispa.cnr.it

**Keywords:** rosemary, extraction procedures, high pressure liquid chromatography–diod-array detection–electrospray ionization–quadrupole–mass spectroscopy (HPLC-DAD-ESI-Q-MS), antioxidant activity, anti-inflammatory activity, NF-κB/MAPK pathway, anti-cancer activity

## Abstract

In this study, we evaluated and compared the chemical composition, the antioxidant, anti-inflammatory, and anti-proliferative effects of four methanol extracts (R1–R4), of *Salvia rosmarinus* Spenn. in two different sites of Southern Italy obtained by maceration or ultrasound-assisted extraction. Extracts of *S. rosmarinus* collected on the Ionian coast are indicated with the abbreviations R1 (maceration) and R2 (ultrasound-assisted extraction). Extracts of *S. rosmarinus* collected on the Tyrrhenian coast are indicated with the abbreviations R3 (maceration) and R4 (ultrasound-assisted extraction). The chemical composition was analyzed using High Pressure liquid chromatography–Diod-Array detection–Electrospray ionization–Quadrupole–Mass Spectroscopy (HPLC-DAD-ESI-Q-MS). The antioxidant activity was analyzed by 2,2′-azino-bis(3-ethylbenzothiazoline-6-sulphonic acid) (ABTS) 2,2-diphenyl-1-picrylhydrazyl (DPPH), β-carotene bleaching, and Ferric Reducing Antioxidant Power (FRAP) assays. Antioxidant features were also assessed in lipopolysaccharide (LPS)-stimulated RAW-264.7 murine macrophages, evaluating Reactive Oxygen Species (ROS) production; in the same experimental model, the anti-inflammatory activity of the extracts was investigated. Interestingly, all extracts displayed antioxidant and anti-inflammatory properties. They exhibited significative nitrite production inhibitory activity, whith IC_50_ values ranging from 3.46 to 5.53 µg/mL, without impairing cell viability. The anti-inflammatory activity was also investigated by Western Blotting and immunofluorescence assay, highlighting the R3 and R4 extracts ability to reduce NF-κB translocation, as well as to disrupt the MAPKs signaling pathway. Extracts exhibited both potential anti-proliferative activity on breast cancer cells, inducing apoptosis, without affecting non-tumorigenic cells, and the ability to inhibit MDA-MB-231 cells’ motility. Finally, the rosemary extracts treatment significantly reduced the power of conditioned media, from MCF-7 or MDA-MB-231 cells to induce nitrite production on RAW 264.7 cells, confirming their promising anti-inflammatory activity.

## 1. Introduction

Cancer is nowadays one of the biggest health problems in the world. In particular, breast cancer is the most common type of carcinoma in women and represents their major cause of death worldwide [1]. Tumor in general, as well as the breast cancer, is characterized by an aberrant growth and ability to invade and metastasize different tissues and organs along the organism [2,3]. Numerous can be the causes that determine the neoplastic event onset, a series of more or less important events that ultimately determine the subversion of the cell that goes from being healthy to a cancer cell [4,5,6]. In recent years a close correlation between oxidative stress and cancer has been observed [7,8]. Indeed, a massive presence of oxygen free radicals [9] can determine important changes in the cell favoring a cancer phenotype onset and, therefore, giving way to the cancer process [10,11]. However, in addition to oxidative stress, the carcinogenesis process may be due to an inflammatory process [12,13]. The inflammatory process is one of the processes that the body sets in motion as protective mechanism against pathogens or even tissue damage in order to facilitate its repair. However, if this process is prolonged over time it causes a chronic inflammation of the tissue which can determine the onset of a neoplastic event [14]. Indeed, there are several cancers associated with inflammatory processes such as colorectal cancer, prostate cancer, and many others [15,16,17].

Given the large number of breast cancer cases diagnosed worldwide and given the different causes and characteristics that distinguish breast cancers, researchers are asked to continuously identify new therapeutic targets and new drugs that can hit this pathology.

As pointed out recently, natural products from medicinal plants represent a fertile ground for the development of novel anti-cancer agents [18,19]. Indeed, natural products very often are less toxic than synthetic products and contain within them various potential activities especially if we refer to the phytocomplex, in which different substances work in concert with each other [20,21].

Based on that, several phytocomplexes have shown in vitro a promising activity against different cancer types [18,22,23] and in oxidative and inflammatory states [18,24,25]. This evidence pushes research to better analyze and use this source for the research of compounds with remarkable biological activity and low toxic activity. Despite the promising biological activity exerted by the various phytocomplexes and their low toxicity towards healthy cells, a limit to their use is due to the possible reduced intestinal absorption and to the probable interactions with the intestinal microbiota [26]. Indeed, to overcame these problems, new carrier vehicles for their delivery are increasingly being studied [27].

*Salvia rosmarinus* Spenn. (Lamiaceae) is one of the most popular plants, commonly known as rosemary. The most used name, *Rosmarinus officinalis*, is considered a synonym of the actual name, *Salvia rosmarinus*, because several investigations evidenced as *Rosmarinus* L. are nested in *Salvia* L. [28]. *S. rosmarinus* is cultivated worldwide but it is native to the Mediterranean region. Rosemary leaves are extensively used traditionally to treat some diseases. In fact, this plant has drawn attention due to its biological activities, including antioxidant, antibacterial, hypoglycaemic, anticancer, hepatoprotective, anti-inflammatory, and antithrombotic effects [29,30,31,32,33]. These biological properties have made rosemary a new potential therapeutic agent for the treatment of many diseases. Furthermore, rosemary has been largely used in the Mediterranean diet and is widely used as a safe and effective natural antioxidant in the food industry. The biological properties of *S. rosmarinus* are principally attributed to the presence of polyphenols, including carnosic acid and rosmarinic acid, and phenolic diterpenes including carnosol [34,35]. While the chemical composition of these secondary metabolites varies considerably depending on ecological conditions, rosemary extracts contain biologically active compounds that make them unique [36].

The current study was planned to assess and to compare the chemical composition, the antioxidant power, the anti-inflammatory, and anti-cancer effects of four Italian *S. rosmarinus* extracts obtained by two extraction procedures and collected in two different localities of southern Italy, when the amount of carnosic acid was negligible.

## 2. Materials and Methods 

### 2.1. Chemicals and Reagents 

Ascorbic acid, propyl gallate, 2,2-diphenyl-1-picrylhydrazyl (DPPH), butylated hydroxytoluene (BHT) β-carotene, and 2,2′-azino-bis(3-ethylbenzothiazoline-6-sulphonic acid) (ABTS), dimethyl sulfoxide (DMSO), and 3-(4,5-Dimethyl-2-thiazolyl)-2,5-diphenyl-2H-tetrazolium bromide (MTT) were acquired from Sigma-Aldrich S.p.a. (Milan, Italy). All solvents used in this study are of analytical grade. LC-MS quality grade acetonitrile and methanol were purchased from Carlo Erba (Val de Reuil, France). Ultrapure water (18.2 MΩ.cm) was obtained from Elga and Purelab Classic (Veolia Water, Antony, France). Formic acid (used at 10 g/L) was obtained from Carlo Erba (Val de Reuil, France). Standard compounds, luteolin-7-glucoside (purity > 98%), rosmarinic acid (purity > 99%), rutin (purity > 99%), carnosol (purity > 90%), carnosic acid (purity > 90%), and ursolic acid (purity > 98%) were purchased from Extrasynthese (Genay, France).

### 2.2. Plant Materials 

The aerial parts of *S. rosmarinus* were harvested in May 2018 in two distinct sites of southern Italy, such as Praia a Mare (Tyrrhenian coast) (Latitude: 39°54′6”84 N, Longitude: 15°46′48”36 E; voucher specimen n. CLU 23974) and in June 2018 in Cirò superiore (Ionian coast) (Latitude 39°23′0”24 N, Longitude: 17°3′48”24 E; voucher specimen n. CLU 23969). Aerial parts of a single rosemary plant in each area were harvested in order to obtain an adequate quantity for the chemical and biological analyses and examined for integrity and absence of dust and insect contamination.

Samples were authenticated by Dr. NG Passalacqua at the Natural History Museum of Calabria and Botanic Garden, University of Calabria (Rende, CS, Italy). 

### 2.3. Extraction Procedure 

The fresh aerial parts of *S. rosmarinus* were exhaustively extracted by using methanol as a solvent by the following procedures: (a) maceration (250 g, 1 L, 3 × 72 h); (b) ultrasound-assisted extraction (50 g, 150 mL, 3 × 1 h) using a Branson 3800 ultrasonic system, series CPXH (130 W, 40 kHz frequency) (Milan, Italy). After being filtered (using Whatman N. 1 filter paper) and combined, extracted solutions were evaporated under reduced pressure in order to obtain dry crude extracts. Extracts were stored at +4 °C in brown bottle until analyzed for their chemical profile and tested for their biological activity. Extracts of *S. rosmarinus* collected on the Ionian coast are indicated with the abbreviations R1 and R2 for maceration and ultrasound-assisted extraction, respectively. Extracts of *S. rosmarinus* collected on the Tyrrhenian coast are indicated with the abbreviations R3 and R4, for maceration and ultrasound-assisted extraction, respectively. 

### 2.4. HPLC-DAD-ESI-Q-MS Profiling

Analyses of the four *S. rosmarinus* extracts were performed using an HPLC-DAD-MS ThermoScientific Dionex U3000 (Thermo-Dionex, Les Ulis, France) including a quaternary pump (LPG-3400 SD), an autosampler thermostat (WPS-3000TSL), a column thermostat (TCC-3000SD) and a Diode Array Detector (DAD-3000) (Thermo-Dionex, Les Ulis, France) on line with a quadripole mass spectrometer (Surveyor MSQ plus System, Thermo-Dionex, Les Ulis, France). The analytical column was a 100 × 2.1 mm i.d. C18 Acclaim Polar advantage II (3 µm, 120 Å), (Dionex Bonded Silica Products, Les Ulis, France) and it was heated to 35 °C during the analyses. Before analyses, all extracts were filtered on 0.2 µm Econo filter Nylon 13 mm 0.2 µm (Agilent, Les Ulis, France). Two chromatographic mobile phases were employed for a gradient elution as follows: eluent-A formic acid (1%, *v*/*v*) in water, eluent-B formic acid (1%, *v*/*v*) in acetonitrile. The binary gradient was formulated as 5% eluent-B during 5 min then in 10 min eluent-B was reached 20%, and in 5 min eluent-B was reached 25%, in 10 min eluent-B was reached 30%, in 10 min eluent-B was reached 40%, in 5 min eluent-B was reached 50%, in 5 min eluent-B was reached 80%, then stay at 80% during 3 min before come back at initial conditions in 2 min. The pump flow rate was 0.5 mL/min and the injection volume was 20 μL. UV spectra were performed using a diode array detector with a wavelength scanning between 200 and 400 nm.

Detection at specific wavelengths 210, 254, 280, and 350 nm, were used to record the chromatograms. The chromatographic effluent carried by a stream of nitrogen was directed into the electrospray ionization source [31] of the mass spectrometer (MS). The MS was operated in the positive and negative ionization modes with the following operating conditions: ion spray voltage 3 kV, curtain gas 50 psi, Q energy was 70 V, cone voltage 50 V, desolvatation temperature 500 °C, and ion energy 0.8 V. In all cases, mass spectra were acquired in the range of 100–1000 Th. Chromeleon^®^, version 6.8 software provided by ThermoScientific Dionex (Les Ulis, France) was used for results’ treatment. All experiments were conducted successively over 3 days, in triplicate each time. Finally, several compounds present in the extracts have been identified and quantified using a methanolic standard solution of compounds previously described in *S. rosmarinus* [37]. The following solutions have been prepared: luteolin-7-glucoside (0.26 g/L), rosmarinic acid (0.97 g/L), rutin (0.16 g/L), carnosol (0.51 g/L), carnosic acid (0.50 g/L), and ursolic acid (0.71 g/L). Calibration curves used a linear fitting (unweight and not forced to axis-origin) in the concentration range 0.008–0.4 g/L. A coefficient determination *R*^2^ > 0.99 were used as acceptability thresholds for calibration purposes. Dry extracts of *S. rosmarinus* (R1–R4) are dissolved in methanol before being analyzed in HPLC-DAD-MS (R1, 2.04 g/L; R2, 1.72 g/L; R3, 1.84 g/L, and R4, 1.72 g/L). Chromatograms recorded of standards after HPLC-DAD-ESI-Q-MS experiments are reported in Supplementary Materials. All experiments were conducted successively over 3 days, in triplicate each time. 

### 2.5. In Vitro Antioxidant Activity 

Several approaches are used to investigate the antioxidant property of plant extracts. Antioxidant activity should not be investigated based on a single antioxidant assay. In fact, in practice various in vitro methods are carried out for evaluating the potential antioxidant effects with the samples of interest. Herein, four tests have been applied to analyze the antioxidant activity of rosemary extracts R1–R4.

#### 2.5.1. DPPH (2,2-diphenyl-1-picrylhydrazyl) Assay

The DPPH radicals’ scavenging activity of *S. rosmarinus* extracts was determined according to the method previously described [38]. A mixture of DPPH methanol solution (800 μL at concentrations of 1.0 × 10^−4^ M), and methanol solutions of rosemary samples (200 μL, at concentrations in the range 1–1000 μg/mL) was prepared. The absorbance was read at 517 nm. 

#### 2.5.2. ABTS (2,2′-azino-bis(3-ethylbenzothiazoline-6-sulphonic acid)) Assay

The ABTS radicals’ scavenging assay measured the ability of an antioxidant to react with ABTS radicals [38]. As with the DPPH assay, the ABTS test is simple, rapid, and versatile with hydrophilic and lipophilic samples. *S. rosmarinus* extracts (10 μL) (at concentrations in the range 1–400 μg/mL) were added to the ABTS solution, and the absorbance was measured after 6 min at 734 nm. 

#### 2.5.3. Ferric Reducing Antioxidant Power (FRAP) Test

The FRAP test measures the ability of antioxidants to reduce ferric iron. In particular, this method estimates the change in absorbance that occurs when the TPTZ (2,4,6-tripyridyl-*s*-triazine)-Fe^3+^ complex is reduced to the TPTZ-Fe^2+^ form in the presence of an antioxidant [39]. *S. rosmarinus* extracts were dissolved in methanol at the concentration of 2.5 mg/mL and tested. The absorbance was measured at 595 nm. 

#### 2.5.4. Carotene Bleaching Test

The ability of *S. rosmarinus* extracts to inhibit lipid peroxidation was examined by using the β-carotene bleaching test [39]. Rosemary extracts were tested at concentrations in the range of 1–100 βg/mL at initial time (t = 0 min), and after 30 and 60 min of incubation, by using propyl gallate as a positive control. 

### 2.6. Cell Cultures

Murine macrophages RAW 264.7 cell line were purchased from the American Culture Collection (ATCC, Manassas, VA, USA) and cultured in DMEM (Sigma, St. Louis, MO, USA) supplemented with 10% Fetal Bovine Serum (FBS, Sigma-Aldrich), 2 mM L-glutamine (Gibco, Life Technologies, Waltham, MA, USA) and 1% penicillin/streptomycin (Gibco, Life Technologies, Waltham, MA, USA). Breast cancer cells (MCF7 and MDA-MB-231) had been purchased from the American Culture Collection (ATCC) and cultured in DMEM/F12 (Sigma-Aldrich, St. Louis, MO, USA) supplemented with 10% FBS, 2 mM l-glutamine (Gibco, Life Technologies), and 1% penicillin/streptomycin (Gibco, Life Technologies, Waltham, MA, USA). MCF-10A cells were cultured as previously reported [18]. All cell lines were cultured at 37 °C in 5% CO_2_ in a humidified atmosphere [40]. Extracts were solubilized in DMSO and the solvent was kept at less than 1%. 

### 2.7. Inhibition of NO Production in LPS-Stimulated RAW 264.7 Cells

Griess reagent was used to determine the presence of nitrites, stable oxidized products of NO in cell culture media [25]. RAW 264.7 cells were seeded in 24-well plates with a density of 2 × 10^5^ cells/well and cultured in complete medium overnight. Cells were then treated, simultaneously, with LPS (1 µg/mL) and different concentrations of extracts for 24 h. DMSO (Sigma-Aldrich, St. Louis, MO, USA) was used as a vehicle control. Then, 100 µL of cell culture supernatant was combined with 100 µL of Griess reagent in a 96-well plate followed by spectrophotometric measurement at 550 nm using a microplate reader.

### 2.8. Immuno-Fluorescence Monitoring Nuclear Factor Kappa B (NF-κB) Translocation

RAW 264.7 cells were seeded on coverslip in 6-well plates at a density of 1 × 10^5^ cells/well and cultured overnight in complete medium. Next, cells were treated for 1 h with LPS (1 µg/mL) and extracts, using their IC_50_ values. After treatment end, cells were fixed with ice cold methanol for 20 min at −20 °C, washed three times for 5 min with Tris buffered saline (TBS, Sigma-Aldrich), and incubated for blocking with 5% bovine serum albumin (BSA, Sigma-Aldrich) in TBS for 40 min at 37 °C. Then, cells were incubated for 40 min at 37 °C in anti-NF-κB p65 monoclonal antibody (Santa Cruz, Biotechnology, Dallas, TX, USA), diluted 1:200, as previously described [41]. Next, they were washed three times for 5 min with TBS to discard the excess of primary antibody, incubated for 40 min at 37 °C in anti-mouse IgG-TRITC (Sigma-Aldrich) diluted 1:300, and subsequently washed three times for 5 min with TBS. Images at 20× magnification were taken on Olympus BX41 microscope with CSV1.14 software, using a CAMXC-30 for image acquisition.

### 2.9. Reactive Oxygen Species Assessment

CM-H_2_DCFDA (ThermoFisher Scientific, Waltham, MA, USA), is a fluorescent dye useful as an indicator for reactive oxygen species [9], as previously described [42,43]. Briefly, 2 × 10^5^ RAW 264.7 cells/well were seeded in 6-well plates and treated with for 1 h with LPS (1 µg/mL) and extracts, using their IC_50_ values. After treatment, cells were washed with PBS, collected, resuspended in 5 μM CM-H_2_DCFDA (ThermoFisher Scientific) in PBS, and incubated 45 min at 37 °C. Stained cells were collected by centrifugation, and resuspended in fresh pre-chilled medium. Finally, fluorescence of samples was quantified with a fluorimeter (Synergy H1 microplate reader, BioTek, Winooski, VT, USA), and fluorescence intensity was normalized by a viable cell number (TC20 automated cell counter, Bio-Rad, Hercules, CA, USA).

### 2.10. Cell Viability Assay

Cell viability was determined as described previously [44] by using 3-(4,5-Dimethyl-2-thiazolyl)- 2,5-diphenyl-2H-tetrazolium bromide (MTT, Sigma-Aldrich, St. Louis, MO, USA) assay. Briefly, RAW 264.7 cells have been treated with different concentrations of compounds for 24 h. In the same way, MCF-7, MDA-MB-231 and MCF-10A cells have been treated with different concentrations of compounds for 72 h. After treatment end, MTT solution was added to each well (to a final concentration of 0.5 mg/mL) and plates were incubated at 37 °C for 2 h until the formation of formazan crystals. DMSO-solubilized formazan in each well was quantified by reading the absorbance at 570 nm using a microplate reader.

### 2.11. Wound-Healing Scratch Assay

MCF-7 and MDA-MB-231 cells were seeded into 6-well plate and cultured overnight in complete medium to assess cell motility by wound-healing scratch assay [18]. Upon confluence, a wound was made by scratching across the monolayer cells on the bottom of the wells by using a p-200 pipette tip followed by treatment with extracts at IC_50_ values for 24 h. Then, cells were fixed and stained with Coomassie brilliant Blue methanol solution. 

Photographs were taken at 4× magnification using phase-contrast microscopy and are representative of three independent experiments. The rate of wound healing was quantified from the picture using Adobe Photoshop software and standard deviations were determined by GraphPad-Prism 8.3.0 software (GraphPad Inc., San Diego, CA, USA).

### 2.12. Conditioned Medium Effects Assessment

Production and collection of conditioned medium was conducted as previously described [45], with some modifications. MCF-7 and MDA-MB-231 cells were seeded into 24-well plate and cultured overnight in complete medium. Upon 70–80% of confluence, cells were treated with rosemary extracts at IC_50_ values, for 24 h. Then, the medium was collected and dispensed into 24-well plate, in which previously RAW 264.7 cells were plated, and incubated for 24 h. Then, 100 µL of cell culture supernatant was combined with 100 µL of Griess reagent in a 96-well plate followed by spectrophotometric measurement at 550 nm using a microplate reader.

### 2.13. TUNEL Assay

Fragmentation of DNA, a late event during apoptosis, was determined by using terminal deoxynucleotidyl transferase-mediated deoxyuridine triphosphate nick end-labeling (TUNEL) assay, based on the enzymatic labelling of DNA strand breaks, using terminal deoxynucleotidyl transferase-mediated deoxyuridine triphosphate nick end-labeling (TUNEL) assay. Labeling was conducted using TUNEL assay kit (Promega, Madison, WI, USA), as previously described [46], on MCF-7 and MDA-MB-231 cells treated for 72 h with DMSO or extracts at IC_50_ values. Nuclear staining was performed by using 0.2 mg/mL 4′,6- diamidino-2-phenylindole (DAPI; Sigma-Aldrich, St. Louis, MO, USA) and samples were analyzed by using a fluorescent microscope (Olympus BX4 (Shinjuku, Tokyo, Japan) with CSV1.14 software, using a CAMXC-30 for image acquisition).

### 2.14. Hemolysis Assay

Fresh human blood from healthy volunteers was collected in sodium citrate tubes and centrifuged at 2000 rpm for 10 min to isolate red blood cells (RBCs) as a pellet, as previously described [47]. RBCs were washed three times with cold PBS pH 7.4 and resuspended in the same buffer (10% *v*/*v*). Further, 60 µg/mL of the different extracts were added to the erythrocyte suspension and incubated for up to 24 h at 37 °C. The release of hemoglobin was determined after centrifugation (2000 rpm for 10 min) by photometric analysis of the supernatant at 540 nm at two different endpoints (1 and 24 h), using a microplate reader (Synergy H1 microplate reader, BioTek). Complete hemolysis was achieved by using 0.1% (*v*/*v*) Triton X-100 which yielded the 100% positive control value while 0.5% DMSO, used as solvent for the extracts, gave the negative control value. 

### 2.15. Statistical Analysis

Data are presented as the mean values ± standard deviation, taken over ≥3 independent experiments, with ≥3 replicates per experiment, unless otherwise stated. Statistical significance was measured by using the analysis of variance (ANOVA) test. A *p* value ≤ 0.05 was considered statistically significant. Non-linear regression analysis (GraphPad Prism 8.3.0 software) was used to generate sigmoidal dose-response curves to calculate the IC_50_ values.

## 3. Results and Discussion

### 3.1. Chemical Composition 

*S. rosmarinus* aerial parts collected in two different areas of southern Italy were herein subjected to two extraction procedures namely maceration and ultrasound-assisted extraction by using methanol as solvent. As evident in Table 1, except for R4, samples exhibited comparable extraction yields. 

**Figure 1:**
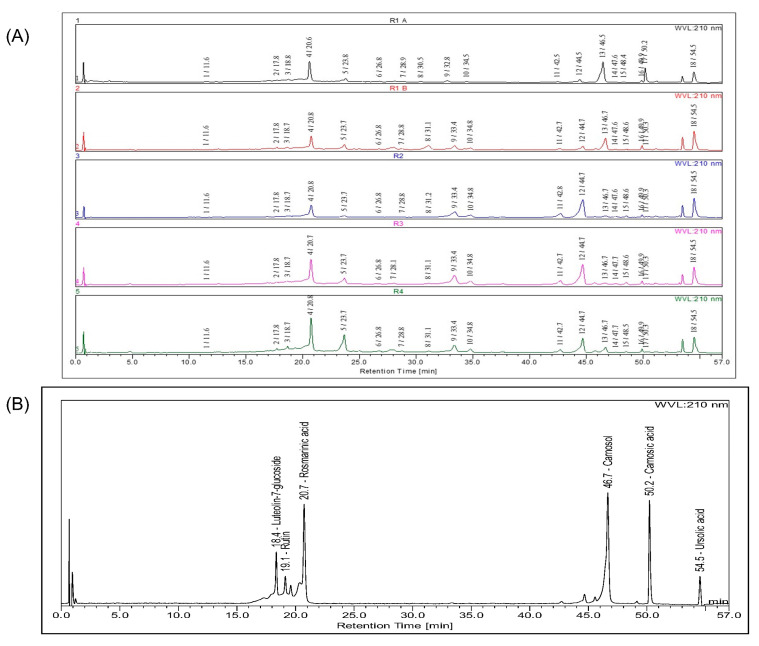
Compounds identification at 210 nm: (**A**) Profiles of *S. rosmarinus* extracts: R1 A t = 1 h, R1 B t = 1d, R2 t = 2d, R3 t = 2d, and R4 t = 2d (t = time between dissolution of the sample and analysis). (**B**) Mixture solution of standard compounds: luteolin-7-glucoside (0.052 g/L), rosmarinic acid (0.194 g/L), rutin (0.032 g/L), carnosol (0.102 g/L), carnosic acid (0.082 g/L), and ursolic acid (0.142 g/L).

The phytochemical compositions of *S. rosmarinus* extracts were determined using an HPLC-DAD-ESI-Q-MS mass spectrometric approach. The identification of rosmarinic acid, luteolin-7-glucoside, rutin, ursolic acid, carnosol, and carnosic acid was sought by authentic standards (Figure 1B). All compounds were identified based on UV spectra, molecular weight (*m*/*z* ion [M+H]^+^ and [M-H]^−^), and bibliographic data concerning the phytochemical composition of *S. rosmarinus* previously published. Chromatograms allowed putative annotation of 18 compounds as reported in Figure 1A and in Table 2.

The results of the quantitative analysis are reported in Table 3 for the four *Rosmarinus* extracts. The commercial control, carnosic acid, and carnosol chromatograms showed several peaks (Figure 1B). These peaks were found in all extracts. The area of these peaks, varying over time, the dosage of these compounds was not carried out.

The results of the quantitative analysis are reported in Table 3 for the four *Rosmarinus* extracts. The commercial controls carnosic acid and carnosol chromatograms showed several peaks (Figure 1B). These peaks were found in all extracts. The area of these peaks varying over time, the dosage of these compounds was not carried out. Eighteen compounds were identified. Fifty-seven compounds have previously been inventoried from a commercial extract of the dried leaves of *S. rosmarinus* [37]. A review listed polyphenolic rosemary compounds [30]. Among them, thirteen phenolic acids have been mentioned, but only rosmarinic acid and caffeic acid are present in our extracts. Among the fifteen flavonoids listed in this publication, only a few flavonoids have been identified in the extracts R1–R4. 

These are hesperidin, isorhamnetin-3-*O*-hexoside, genkwanin, and kaempferol. Studied extracts are rich in phenolic terpenes, in fact among the twelve previously described, eight of them were found in all of our extracts namely carnosic acid, 12-*O*-methylcarnosic acid, carnosol, three rosmanol isomers (epirosmanol, epiisorosmanol, and isorosmanol), rosmaridiphenol, and ursolic acid.

The presence of dihydroxydimethoxy flavone, hispidulin-7-glucoside, luteolin -3′-acetyl-*O*-glucuronide and two rosmanolmethylether isomers have been confirmed by the work of Mena et al. [37].

Quantitative analyses of the R1–R4 extracts in triplicate over three days, showed similar profiles, and areas of invariable chromatographic peaks proving good stability of the main compounds. However, it should be noted that the concentration of carnosol and carnosic acid in the standard solutions as well as the solutions of all the extracts decreased during the analyses. 

With regard to triterpenic acids, ursulic acid, and oleanolic acid, as they do not absorb in UV/Vis, their specific coefficients are similar and it can then be considered that aera under the chromatographic peak at Rt 54 mn corresponds either to the sum of the injected amounts of these compounds. Thus, for the determination of the contents of triterpenic acids, the calibration line constructed with the ursulic acid alone can be used.

Analyzing results reported in Table 3, *S. rosmarinus* extracts from the Ionian side (R1 and R2) seem richer in rosmarinic acid (values of 3.84% and 5.39%, respectively) and in triterpene acids (values of 16.67% and 22.09%) than the extracts from the Tyrrhenian side (R3 and R4) (values of 2.09%, and 2.37%, for rosmarinic acid, respectively, and 21.36% and 9.71% for triterpene acids, respectively).

### 3.2. In Vitro Antioxidant Activity

*S. rosmarinus* extracts were investigated in vitro for their potential antioxidant activity. Data are reported in Table 4. The analysis of results highlights that rosemary extracts from Ionian coast are more active than rosemary extracts from Tyrrhenian coast with some exception. 

In particular, the best radical scavenging power was observed with sample R2 with IC_50_ values of 0.94 and 8.80 μg/mL in an ABTS and DPPH test, respectively. Interesting results were obtained also in a β-carotene bleaching test with sample R2 with IC_50_ values of 7.18 and 6.53 μg/mL after 30 min and 60 min of incubation, respectively. 

Conversely, sample R3 such as extract obtained by maceration of *S. rosmarinus* from Cirò superiore was the most active in FRAP test with a value of 97.20 μM Fe (II)/g.

### 3.3. Nitric Oxide Production in RAW 264.7 Cells

The relationship between oxidative stress and inflammation has been already reported [48], indeed various inflammatory stimuli, such as LPS, were able to initiate different chronic inflammatory diseases by modulating Nitric Oxide (NO) production [49]. NO is synthesized by the enzymatic activity of the inducible Nitric Oxide synthase (*i*NOS) and is believed to be a key mediator of the inflammatory pathway in LPS-stimulated cells. Based on that, we next asked if our rosemary extracts could also act as anti-inflammatory agents. For this purpose, we investigated their ability to affect NO production by testing increasing concentrations of them, up to 60 µM (from 10 µM), on LPS-stimulated RAW 264.7 cells, murine macrophages widely used as an in vitro model to study inflammatory pathways. Firstly, we assessed if the extracts could influence cell viability; the results displayed the different cytotoxicity behavior of them, related to their origin place. In particular, the extracts from samples collected on the Ionian coast (R1–R2) evidenced a slightly more cytotoxic effect compared to those of the R3–R4 extracts from the Tyrrhenian coast (Figure S1). Then, by using the four extracts, at non-toxic concentrations, we evaluated their inhibitory effect on NO production, and we found that all extracts are active, and particularly, R2 and R3 displayed the best activity (Figure S1 and Table 5). 

These findings are in agreement with literature data reporting similar anti-inflammatory properties of *S. rosmarinus* and its bioactive components, when tested in our same experimental model [50]. In addition, our results highlighted that diversity in terms of cytotoxicity and inhibitory effect on NO production, evidenced by different extracts, reflects their different origin. However, the similar behavior on NO production, evidenced for R2 and R3 extracts, belonging to plants from two different origin places and resulted by different extraction procedures, could be related to the same chemical composition of two extracts. Indeed, they are characterized by large amount of carnosic acid (CA) derivatives, widely known as anti-inflammatory agents.

### 3.4. Rosemary Extracts Exert Anti-Inflammatory Effects by Reducing NF-κB Nuclear Translocation and Disrupting the MAPK/NF-κB Pathway

**Figure 2:**
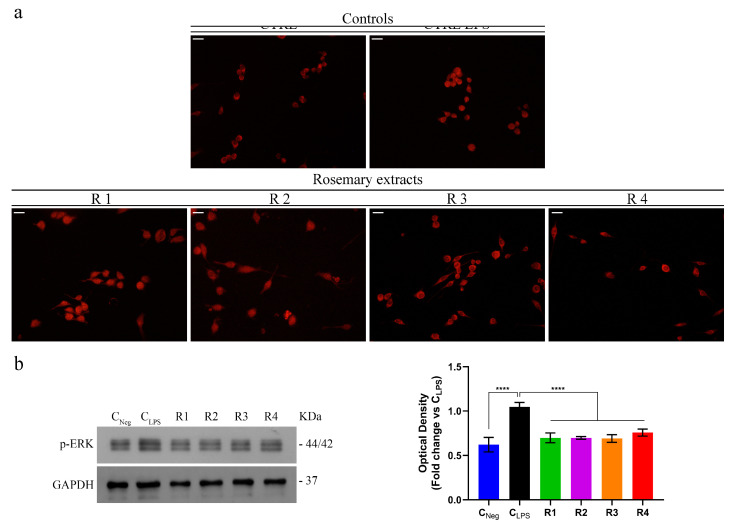
Effects of *S. rosmarinus* extracts on both NF-κB nuclear translocation and mitogen-activated protein kinases (MAPK)/NF-*κB* pathway. (**a**) Immuno-fluorescent localization of NF-κB in RAW 264.7 cells treated for 1 h with dimethyl sulfoxide (DMSO) or 1 µg/mL Lipopolysaccharide (LPS) + DMSO (Control (CTRL) and CTRL LPS, respectively), 1 µg/mL LPS + R1–R4 extracts (at their IC_50_ values, respectively). Scale bar: 50 µm. (**b**) Immunoblotting analysis of p-ERK and relative quantification of expression levels. Glyceraldehyde 3-phosphate dehydrogenase (GAPDH) was used as loading control. Values represent mean ± S.D. of three independent experiments, each one performed with triplicate samples. *p* values were calculated against C_LPS_; **** *p* < 0.001.

It is known that the inducible Nitric Oxide Synthase (*i*NOS) is an important target of the NF-κB transcription factor playing a critical role in inflammatory signal transduction [51,52]. Indeed, both the enzyme expression level and NO production are mediated by it [49,53]. In normal conditions, NF-κB is localized into the cytoplasm, while, in inflammatory responses, it is translocated into the nucleus where it binds to the promoter sequences of several pro-inflammatory genes, including *i*NOS [49,53]. Considering the effects exerted by the rosemary extracts on NO production in LPS-stimulated RAW 264.7 cells, we tested their ability to reduce the NF-κB activation, by monitoring its translocation into the nucleus. The results obtained (Figure 2a) show that R3 and R4 extracts significantly inhibited nucleus translocation of the NF-κB, in respect to that assessed in LPS-stimulated RAW 264.7 cells. Conversely, R1 and R2 extracts were not able to act on the NF-κB translocation suggesting that the reduction in NO production, previously observed, is due to a different mechanism that does not involve the NF-κB-mediated signaling pathway. 

It has been widely reported that, during the inflammatory process of LPS-stimulated macrophages, nuclear factor-κB (NF-κB) activation, is related to that of mitogen-activated protein kinases (MAPKs) pathway [53]. MAPKs, together with NF-κB, trigger transcription of different inflammatory genes, modulating consequently the amplification and propagation of inflammatory-mediated stress signal [49].

The MAPKs pathway comprises several proteins, including extracellular signal-regulated kinase 1/2 (ERK1/2), p38, and c-Jun N-terminal kinase (JNK) [54]. Furthermore, it has been demonstrated that *S. rosmarinus* extracts can play beneficial effects on intestinal inflammation through NF-κB/MAPKs signaling pathway [55]. In this context, in order to investigate the molecular mechanisms that underly R1–R4 extracts’ behavior, by Immunoblotting we assessed if they could play an inhibitory effect on LPS-induced phosphorylation of ERK1/2, the main intermediate proteins of the pathway. The results displayed that the phosphorylation levels of ERK1/2 were increased by LPS stimulation of RAW 264.7 macrophage cells, (Figure 2b). However, the treatment with R1–R4 extracts significantly decreased LPS-induced phosphorylation of ERK1/2 (Figure 2b). Our findings highlighted the R3 and R4 extracts’ ability to significantly reduce the translocation of NF-κB into the nucleus, as well as the power of all the *S. rosmarinus* extracts tested to disrupt the MAPKs signal transduction pathway. 

### 3.5. Rosemary Extracts Exerts Anti-Oxidant Effects by Reducing ROS Levels

**Figure 3:**
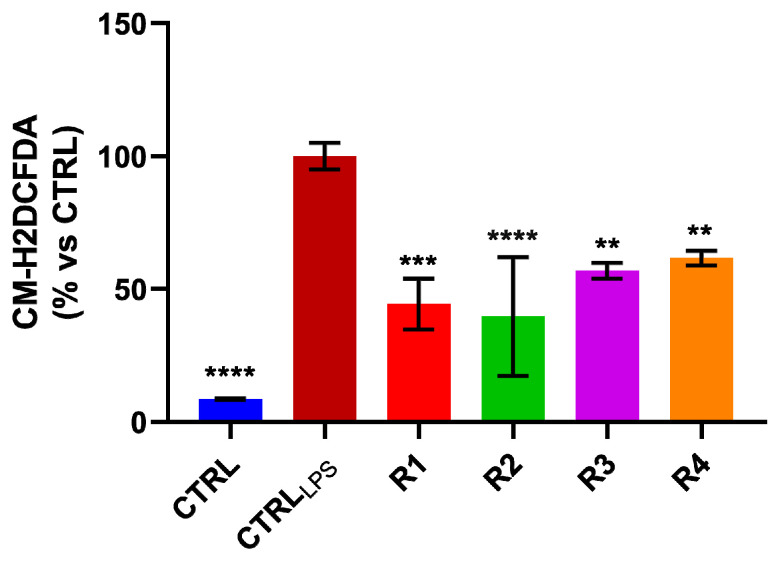
Rosemary extracts reduce Reactive Oxygen Species (ROS) levels in Lipopolysaccharide (LPS)-stimulated RAW 264.7 cells. ROS intracellular levels were measured after rosemary extracts (R1–R4) treatment of LPS-stimulated RAW 264.7 cells, for 24 h. *p* values were calculated against CTRL_LPS_; ** *p* value < 0.01; *** *p* value < 0.001; **** *p* value < 0.0001.

Since inflammation is closely related to oxidative stress and based on our previous findings that showed in vitro both anti-oxidant activity and an inhibitory effect on NO production of the rosemary extracts, we investigated if they could be able to regulate the reactive oxygen species [9] levels, in LPS-stimulated RAW 264.7 cells. In detail, LPS-stimulated RAW 264.7 cells were treated for 24 h, with R1–R4 extracts, at IC_50_ values. We proved that treatment significantly reduces ROS levels (Figure 3), accordingly to the results obtained in DPPH and ABTS assays and opening new scenarios for these extracts as radical scavengers.

### 3.6. Rosemary Extracts Showed a Promising Anti-Proliferative Effect on Breast Cancer Cell Lines

Nowadays, the relationships among cellular oxidative stress, inflammation pathways, and cancer formation and propagation are well-known [50,56]. Moreover, having established that the treatment with rosemary extracts R1–R4 significantly disrupted the MAPKs pathway which is known to regulate cell events such as growth, differentiation, survival, and apoptosis [53], we then assessed the impact of the extracts on cancer cell viability. To this end two different breast cancer cell line, MCF-7 (ERα +) and MDA-MB-231 (triple negative) were plated for MTT assay in the presence of increasing concentrations (10, 20, 40, and 60 µg/mL) of extracts or vehicle alone (Control), for 72 h.

We found that all extracts concentration-dependently inhibited cell growth in both tested breast cancer cells (Figure S2), in particular in MDA-MB-231, with IC_50_ ranging from 6.83 µg/mL to 15.67 µg/mL (Table 6). These values are in agreement with the American National Cancer Institute criteria according to which the IC_50_ limit to consider a crude extract as promising anticancer agent should be lower than 30 µg/mL [57].

The anti-proliferative activity of rosemary extracts in MCF-7 cell lines was also interesting, showing an IC_50_ value ranging from 10.96 to 32.17 µg/mL. Remarkably, anti-proliferative activities of extracts were found to be specific for cancer cells, indeed, their IC_50_ values in non-tumorigenic MCF-10A cell lines were higher rather than those found in all the tested cancer cell lines (Table 6 and Figure S2). Moreover, our findings are in complete agreement with literature data reporting anti-proliferative effects of different species of *S. rosmarinus* genus and highlighting the usefulness of this genus as a source of extracts with potential anticancer activity [58,59].

### 3.7. Rosemary Extracts Trigger Cell Death by Apoptosis in Breast Cancer Cells

Several reports demonstrated that rosemary extracts are able to trigger cell death by apoptosis in different human cancer cell lines. In particular, their polyphenolic compounds such as carnosic acid and carnosol induce apoptosis in PC3, DU-145, and LNCAP prostate cancer cells [60]. Similar results were evidenced on colon and pancreatic cancer cells; in this context, colon SW620 and DLD-1 cancer cells might be more sensitive, than PANC-1 and MIA-PaCa-2 pancreatic cancer cells, to the apoptotic effects of the rosemary extracts [61]. Interestingly, rosemary extract treatment induces apoptosis on the human ovarian cancer cell line A2780, as well as on the cisplatin resistant cell line A2780CP70, by modulating the expression levels of several genes involved in the pathway [62].

Taken together literature data prove that RE induce apoptosis in a wide range (10–100 µg/mL) of IC_50_ values, depending on the different extraction methods used to prepare the rosemary extract [58]. Particularly, a supercritical fluid rosemary extract dose-dependently induces apoptosis in breast cancer cells from different tumor subtypes including T47D and SK-BR-3 cell lines [63].

**Figure 4:**
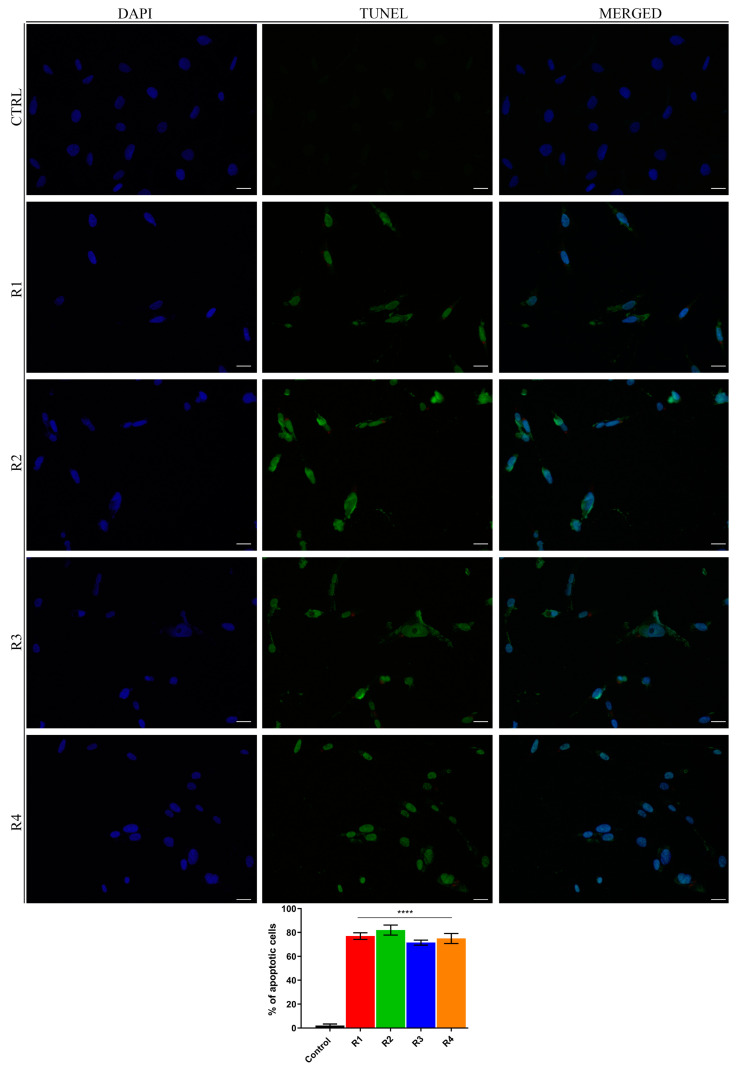
*S. rosmarinus* extracts induce cell apoptotic death. TdT-mediated dUTP nick-end-labeling (TUNEL) assay in MCF-7 cells treated for 72 h with vehicle (Control) or rosemary extracts (R1–R4). The 4′,6- diamidino-2-phenylindole (DAPI) was used for DNA staining, scale bar: 50 μm. Histograms represent means ± S.D. of apoptotic vs control cells from three independent experiments performed in triplicate. *p* values were calculated against control; **** *p* value < 0.0001

On this basis and considering the impact of rosemary extracts tested in this study on MCF-7 and MDA-MB-231 cell viability, we examined if the anti-proliferative activity displayed by them could be due to apoptosis induction. Since DNA fragmentation is a late event in the apoptotic cascade [64], we evaluated this process on both MCF-7 (Figure 4) or MDA-MB-231 (Figure S3) cells after 72 h of R1–R4 extracts exposure, by TUNEL assay. The results revealed that the percentage of TUNEL-positive cells significantly increased in both cell lines, with respect to control (DMSO-treated) cells.

### 3.8. Rosemary Reduces MDA-MB-231 Cells Motility and Pro-Inflammatory Behavior of Breast Cancer Cells

**Figure 5:**
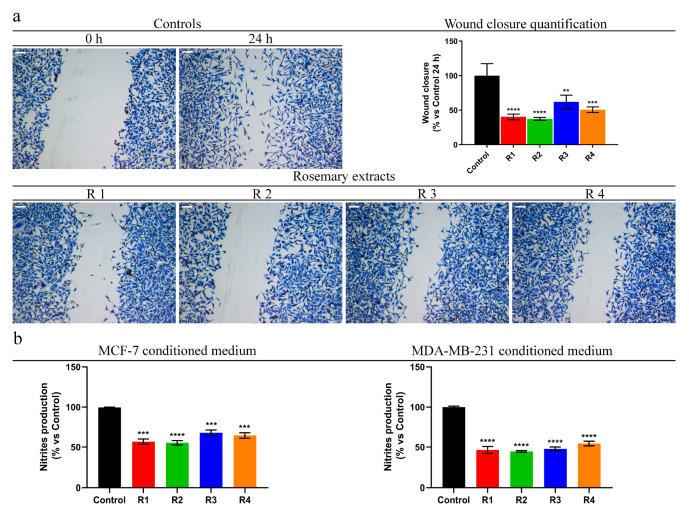
Rosemary extracts (R1–R4) reduce MDA-MB-231 cell motility and pro-inflammatory effects of MCF-7 and MDA-MB-231 conditioned medium. (**a**) Scratch assay on MDA-MB-231 cells treated with dimethyl sulfoxide (DMSO; Control), rosemary extracts (as indicated) at IC_50_ value. Scale bars 50 μm. Histograms represent the relative percentage of wound closure from three different experiments, each performed with triplicate samples. (**b**) Nitrites production assessments after treatment of RAW 264.7 cell line with conditioned medium of MCF-7 or MDA-MB-231 (previously treated with IC_50_ values of rosemary extracts for 24 h) for 24 h. *p* value were calculated against Control; ** *p* value < 0.01; *** *p* value < 0.001; **** *p* value < 0.0001.

Based on the anti-proliferative activity showed by rosemary extracts in the highly invasive and metastatic MDA-MB-231, we next asked if they are also able to exert anti-migratory effects. Cell migration is a very unfavorable feature, because it triggers cancer dissemination and initiates metastatic events [65]. The effect of each extract on motility was performed evaluating the ability of the MDA-MB-231 cells to invade the wound area by wound healing scratch assay. The results showed that, after 24 h of rosemary extracts (R1–R4) exposure, the percentage of wound closure of these cells was significantly reduced when compared to that of the vehicle-treated cells (DMSO) used as a control (Figure 5a). This evidence underlines the potential ability of the tested extracts to decrease the cell motility, and thus could counteract the metastatic capability of this cancer cell line, which still remains the main cause for therapy failure.

During the last decades, different studies established an interesting correlation between inflammation and cancer progression; many cancers are characterized by an etiopathogenesis linked to chronic inflammatory processes [14]. Moreover, tumor cells during their growth release, in the tumor microenvironment, pro-inflammatory mediators which attract leukocytes and modulate the behavior of tumor cells. In the solid tumor microenvironment the inflammatory component may involve different leukocyte population; however, macrophages are the most abundant inflammatory cells, which can enhance the invasiveness of breast cancer [66,67]. In this context, the effect of each rosemary extract on the pro-inflammatory behavior of breast cancer cells was evaluated. In particular, by Griess assay, we monitored the ability of conditioned media (CM), from MCF-7 or MDA-MB-231 cells, exposed for 24 h to R1–R4 extracts, to determine NO production on RAW 264.7 cells. The results showed that the treatment significantly reduces the ability of CM to induce NO production on RAW 264.7 cells, with respect to that of CM from control cells (DMSO-treated cells) (Figure 5b).

### 3.9. Rosemary Extracts Did Not Exert Hemolytic Effects on Peripheral Blood

Finally, in order to evaluate the translational potential of the studied rosemary extracts (R1–R4), we assessed their behavior in human blood by a hemolysis test. This test is commonly used in toxicity studies of interesting plant extracts in the medical field [47].

Due to that, the test was performed by using peripheral blood from healthy volunteer. The results, shown in Figure S4, highlighted the non-hemolytic feature of all the extracts, with acceptable hemolysis rate up to the maximum concentration tested (60 μg/mL).

## 4. Conclusions

Bioactive compounds isolated from natural products play a dominant role as potential drugs with utility in many diseases [59]. Herein we evaluated and compared the biological effects of four extracts of *S. rosmarinus* (R1–R4) collected in two distinct sites of Southern Italy. The extraction was performed by two different procedures, the maceration (extracts R1 and R3) and ultrasound-assisted extraction (extracts R2 and R4). For a long time, *S. rosmarinus* has been used in traditional medicine for attending to several diseases. Recently, literature data established that its extracts are endowed with antioxidant, anti-inflammatory, and anticancer properties [68].

Our findings evidenced that the four extracts tested are provided with antioxidant properties, although rosemary extracts from Ionian coast are more active than those from Tyrrhenian coast.

Additionally, we found that R1–R4 extracts treatment significantly reduced ROS levels, in LPS-stimulated RAW 264.7 cells, according to their antioxidant activity and proposing these extracts as radical-scavenger candidates. Based on literature data indicating that antioxidant properties are related to anti-inflammatory behavior [48], we established that all extracts are also endowed with anti-inflammatory activity as evidenced by a decrease in NO production. However, only R3–R4 extracts exert this effect involving the NF-κB-mediated signaling pathway.

In the same way, the different behavior of the extracts concerning inhibitory effect on NO production is sometimes related to their different origin, rather than different extraction procedure used. This result prompt us to assert that ultrasound-assisted extraction is a very quick process, alternative to the maceration procedure, able to produce cleaner bioactive *S. rosmarinus* leaf extracts. Furthermore, according to the ability of *S. rosmarinus* extracts to disrupt the MAPKs signal transduction pathway, which is known to regulate cell events such as growth, differentiation, survival, and apoptosis [39], we demonstrated that *S. rosmarinus* R1–R4, although poor in carnosic acid, are able to exert anti-proliferative activity in MCF-7 and MDA-MB-231 breast cancer cells and to trigger cell death by apoptosis.

Interestingly, we found that the studied extracts can reduce both pro-inflammatory behavior and motility of MDA-MB-231 cells, a highly invasive breast cancer cell line, evidencing their potential ability to counteract the metastatic ability of this cancer cell line, which yet remains the main cause for therapy failing. Noteworthy, our findings evidenced a lack of toxic effects on red blood cells, thus supporting a safe use of the extracts in in vivo experimental models. However, the several biological activities (such as antioxidant and cytotoxic activities) exerted by rosemary extracts and clearly explained in this report highlight the *S. rosmarinus* as an interesting source of biologically active compounds. At the same time, more studies are needed to better understand their intestinal absorption. In this context, numerous studies have been developed [69,70,71], although most of them have been focused on carnosic acid, which is contemplate the most bioactive compound in rosemary extracts [72,73]. Recent evidence highlighted that polyphenols in a rosemary infusion were partly absorbed and widely metabolized in both HepG2 and Caco-2 cells, as in vitro models of liver and intestine, respectively [74]. Additionally, it will be needful to investigate on the intestinal microbiota activity which could limit phytocomplexes’ effects. Indeed, some reports demonstrated that human microbiota are able to meddle with both bioavailability and bioactivity of phytocomplexes.

Phytocomplexes can be metabolized by the microbiota, but they can also modulate it, in order to improve their role in the prevention and amelioration of several diseases [75]. Moreover, the presence of several classes of compounds identified in all the extracts tested underlines the need to further analyze the effects of their single bioactive components.

## Figures and Tables

**Table 1:** Site of collection, extraction procedure, and extraction yield (%) of *S. rosmarinus* samples.

*S. rosmarinus*	Site of Collection (Southern Italy)	Extraction Procedure	Yield (%)
R1	Cirò Superiore (Ionian coast)	Maceration	11.63 ± 1.34
R2		Ultrasound-assisted extraxction	10.45 ± 1.11
R3	Praia a Mare (Thyrrenian coast)	Maceration	10.71 ± 1.43
R4		Ultrasound-assisted extraxction	7.44 ± 0.87

Data are expressed as mean ± S.D. (*n* = 3).

**Table 2:** Retention time (Rt), wavelengths of maximum absorption in the UV–VIS region at 210 nm, mass spectra data, and compounds identification in *S. rosmarinus* extracts.

Peak	Rt	UV λ(nm)	Molecular Ion [M−H]^−^ (*m*/*z*)	Molecular Ion [M+H]^+^ (*m*/*z*)	Identification	Ref.
1	11.6	220/240/295/325	179		Caffeic acid	[30]
2	17.8	200/225/285	609	611	Hesperidin	[30,37]
3	18.8	208/275/340	477	479	Isorhamnetin-3-*O*-hexoside	[30,37]
4	20.6	200/280/290/330	359	361	Rosmarinic acid (cis,trans) ^2^	[30]
5	23.8	205/242/270/340	461	463	Hispidulin-7-glucoside	[37]
6	26.8	205/270/340	503	505	Luteolin -3′-acetyl-*O*-glucuronide	[37]
7	28.9	208/286	345	347	Rosmanol isomer ^1^	[30,37]
8	30.4	205/285	345	347	Rosmanol isomer ^1^	[30,37]
9	32.8	205/290	345	347	Rosmanol isomer ^1^	[30,37]
10	34.5	205/290		315	Dihydroxydimethoxy flavone	[37]
11	42.5	208/280/335	283	285	Genkwanin	[30,37]
12	44.4	207/291	359		Rosmanol methylether isomer	[37]
13	46.5	205/286	329	331	Carnosol ^2^	[30]
14	47.6	265/300/345	593		Kaempferol	[30]
15	48.4	227/280	315	317	Rosmaridiphenol	[30]
16	49.9	208/233/285	345		12-*O*-methylcarnosic acid	[30,37]
17	50.1	204/235/287	331		Carnosic acid ^2^	[30,37]
18	54.5			593 ^3^	Oleanolic acid/Ursolic acid ^2^	[30]

^1^ Rosmanol, epirosmanol, epiisorosmanol or/and isorosmanol. ^2^ Identification confirmed using commercial standards. ^3^ Sodium and formic adduct. Rt: retention time.

**Table 3:** Rosmarinic acid and triterpene acids quantification in dry extracts R1–R4 by HPLC-DAD-Q-MS.

Extract	Rosmarinic Acid (Rt 20.6 min)		Triterpene Acids (Rt 54.5 min)	
	(*)	(% g/g)	(*)	(% g/g)
R1	0.11 ± 0.02	5.39	0.340 ± 0.008	16.67
R2	0.066 ± 0.009	3.84	0.380 ± 0.023	22.09
R3	0.0384 ± 0.0067	2.09	0.393 ± 0.043	21.36
R4	0.0407 ± 0.0068	2.37	0.167 ± 0.026	9.71

(*) Results are expressed as means values (g/L) + standard deviation (*n* = 6). Rt: retention time.

**Table 4:** In vitro antioxidant activity of *S. rosmarinus* extracts.

*S. rosmarinus*	DPPH ^a^ Test IC_50_ (μg/mL)	ABTS ^b^ Test IC_50_ (μg/mL)	β-carotene Bleaching Test IC_50_ (μg/mL)		FRAP ^c^ Test μM Fe (II)/g ^1^
			30 min	60 min	
R1	8.83 ± 0.82 **	1.48 ± 0.10	12.53 ± 1.20 ****	8.87 ± 0.85 ****	95.17 ± 6.62
R2	8.80 ± 0.81 **	0.94 ± 0.09	7.18 ± 0.76 ****	6.53 ± 0.65 ****	80.09 ± 5.01
R3	11.23 ± 1.14 ****	1.57 ± 0.14 ***	45.57 ± 3.90 ****	26.61 ± 2.62 ****	97.20 ± 6.30
R4	14.76 ± 1.42 ****	2.01 ± 0.24	10.84 ± 1.05 ****	10.75 ± 1.03 ****	76.77 ± 5.13
Positive control					
Ascorbic acid	5.02 ± 0.80	1.71 ± 0.06			
Propyl gallate			0.09 ± 0.004	0.09 ± 0.004	
BHT					63.20 ± 4.32

Data are expressed as mean ± S.D. (*n* = 3). ^1^: tested at concentration 2.5 mg/mL. Differences within and between groups were evaluated by one-way analysis of variance test followed by a multicomparison Dunnett’s test α = 0.05 compared with the positive controls **** *p* < 0.0001, *** *p* <0.001, ** *p* < 0.01. ^a^ 2-diphenyl-1-picrylhydrazyl. ^b^ 2,2′-azino-bis(3-ethylbenzothiazoline-6-sulphonic acid. ^c^ Ferric Reducing Antioxidant Power.

**Table 5:** Nitrites production inhibitory activity (IC_50_ values).

IC_50_ ± SD (µg/mL)			
R1	R2	R3	R4
3.46 ± 0.78	1.71 ± 0.40	2.03 ± 0.20	5.53 ± 1.26

**Table 6:** Cytotoxic activity of *S. rosmarinus* extracts ^a^.

Cell Line		R1	R2	R3	R4
MCF-7	IC_50_ (µg/mL)	11.48	10.96	23.33	32.17
	95% confidence interval	8.688 to 14.90	8.391 to 14.06	17.07 to 31.77	27.43 to 37.72
MDA-MB-231	IC_50_ (µg/mL)	8.648	6.830	12.24	15.67
	95% confidence interval	5.687 to 12.50	4.169 to 10.31	7.992 to 17.94	12.80 to 19.09
MCF-10A	IC_50_ (µg/mL)	285.6	265.7	241.9	212.3
	95% confidence interval	140.1 to 1456	137.4 to 987.8	140.1 to 585.9	93.64 to 1601

^a^ Data are presented as IC_50_ values (µg/mL) and 95% confidence intervals obtained by nonlinear regression analysis of three independent experiments.

## Supplementary Materials

The following are available online at https://www.mdpi.com/2076-3921/9/9/826/s1, Figure S1: *S. rosmarinus* extracts (R1–R4) modulate nitric oxide (NO) production. Cell viability and nitrites production assessments after treatment of LPS-stimulated RAW 264.7 cell line with different concentration of extracts (as indicated) for 24 h. MTT assay results are expressed as percentage of cell viability versus Control; Griess assay results are expressed as percentage of nitrites production versus Control. Values represent mean ± S.D. of three independent experiments, each one performed with triplicate samples. *p* value were calculated against Control; *** *p* < 0.005; **** *p* < 0.001. Figure S2: Effect of rosemary extracts (R1–R4) treatment on cell viability. Cell growth assessment after treatment for 72 h of MCF-7, MDA-MB-231 and MCF-10A cell lines, using different concentrations (from 10 to 60 μg/mL) of R1–R4, as indicated. Results were quantified by MTT assay and expressed as percentage of growth vs control (cells treated with DMSO, Control). Values represent means ± S.D. of three independent experiments, each performed with triplicate samples. *p* value were calculated against Control; * *p* value < 0.05; **** *p* value < 0.0001. Figure S3: *S. rosmarinus* extracts induce cell apoptotic death. TdT-mediated dUTP nick-end-labeling (TUNEL) assay in MDA-MB-231 cells treated for 72 h with vehicle (Control) or rosemary extracts (R1–R4). DAPI was used for DNA staining, scale bar: 50 μm. Histograms represent means ± S.D. of apoptotic vs control cells from three independent experiments performed in triplicate. *p* value were calculated against Control; **** *p* value < 0.0001. Figure S4: Rosemary extracts did not exert haemolytic effects. Haemolysis assay on RBCs treated with DMSO (Control), 0.1% Tween-100 or rosemary extracts (R1–R4), for 1 h or 24 h. Histograms represent the relative percentage of hemolysis from three different experiments, each performed with triplicate samples. *p* value were calculated against 0.1% Tween-100; **** *p* value < 0.0001. Figure S5. Standard compounds: luteolin-7-glucoside (0.26 g/L), rosmarinic acid (0.97 g/L), rutin (0.16 g/L), carnosol (0.51 g/L), carnosic acid (0.50 g/L), and ursolic acid (0.71 g/L). Figure S6. Standard compounds spectra. Figure S7. Sample compounds spectra.
