# Supplementary material for: Chemical Profile, Antioxidant, Anti-Inflammatory, and Anti-Cancer Effects of Italian Salvia rosmarinus Spenn. Methanol Leaves Extracts
Source: Antioxidants (Basel). 2020 Sep 3;9(9):826. doi: 10.3390/antiox9090826 (PMC7556042; doi:10.3390/antiox9090826)
Supplement: Supplementary file 1 [file antioxidants-09-00826-s001.pdf]

Supplementary Materials

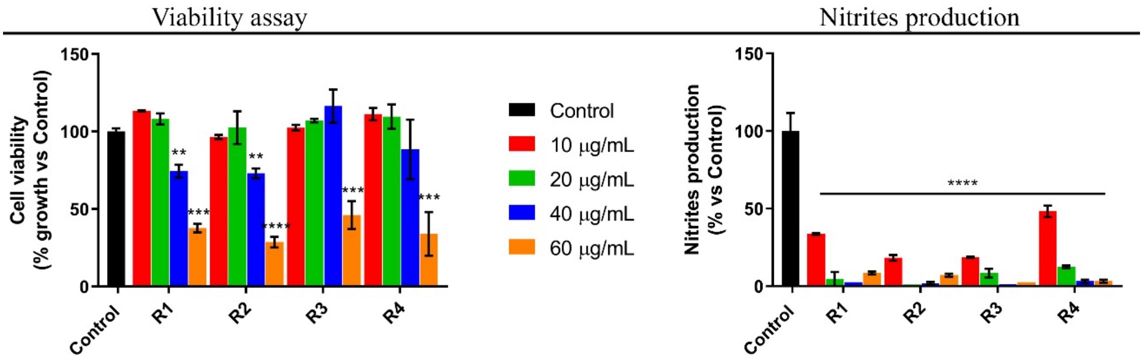

Figure S1. *S. rosmarinus* extracts (R1-R4) modulate nitric oxide (NO) production. Cell viability and nitrites production assessments after treatment of LPS-stimulated RAW 264.7 cell line with different concentration of extracts (as indicated) for 24 h. MTT assay results are expressed as percentage of cell viability *versus* Control; Griess assay results are expressed as percentage of nitrites production *versus* Control. Values represent mean ± S.D. of three independent experiments, each one performed with triplicate samples. *P* value were calculated against Control; *** $p < 0.005$; **** $p < 0.001$.

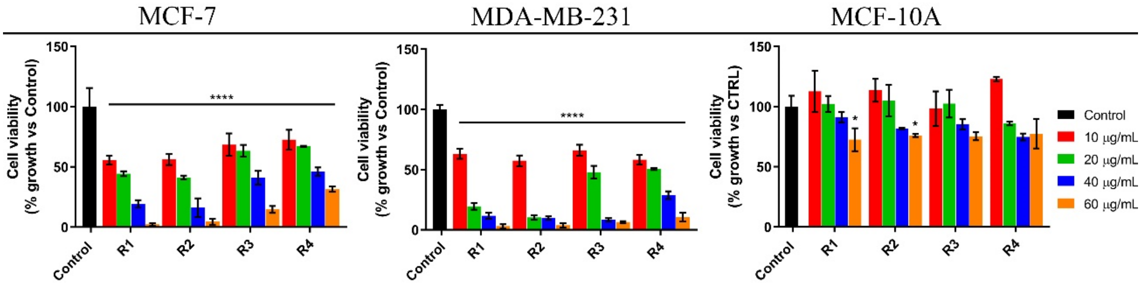

Figure S2. Effect of rosemary extracts (R1-R4) treatment on cell viability. Cell growth assessment after treatment for 72 h of MCF-7, MDA-MB-231 and MCF-10A cell lines, using different concentrations (from 10 to 60 μg/mL) of R1-R4, as indicated. Results were quantified by MTT assay and expressed as percentage of growth vs control (cells treated with DMSO, Control). Values represent means ± S.D. of three independent experiments, each performed with triplicate samples. *P* value were calculated against Control; * P value < 0.05; **** P value < 0.0001.

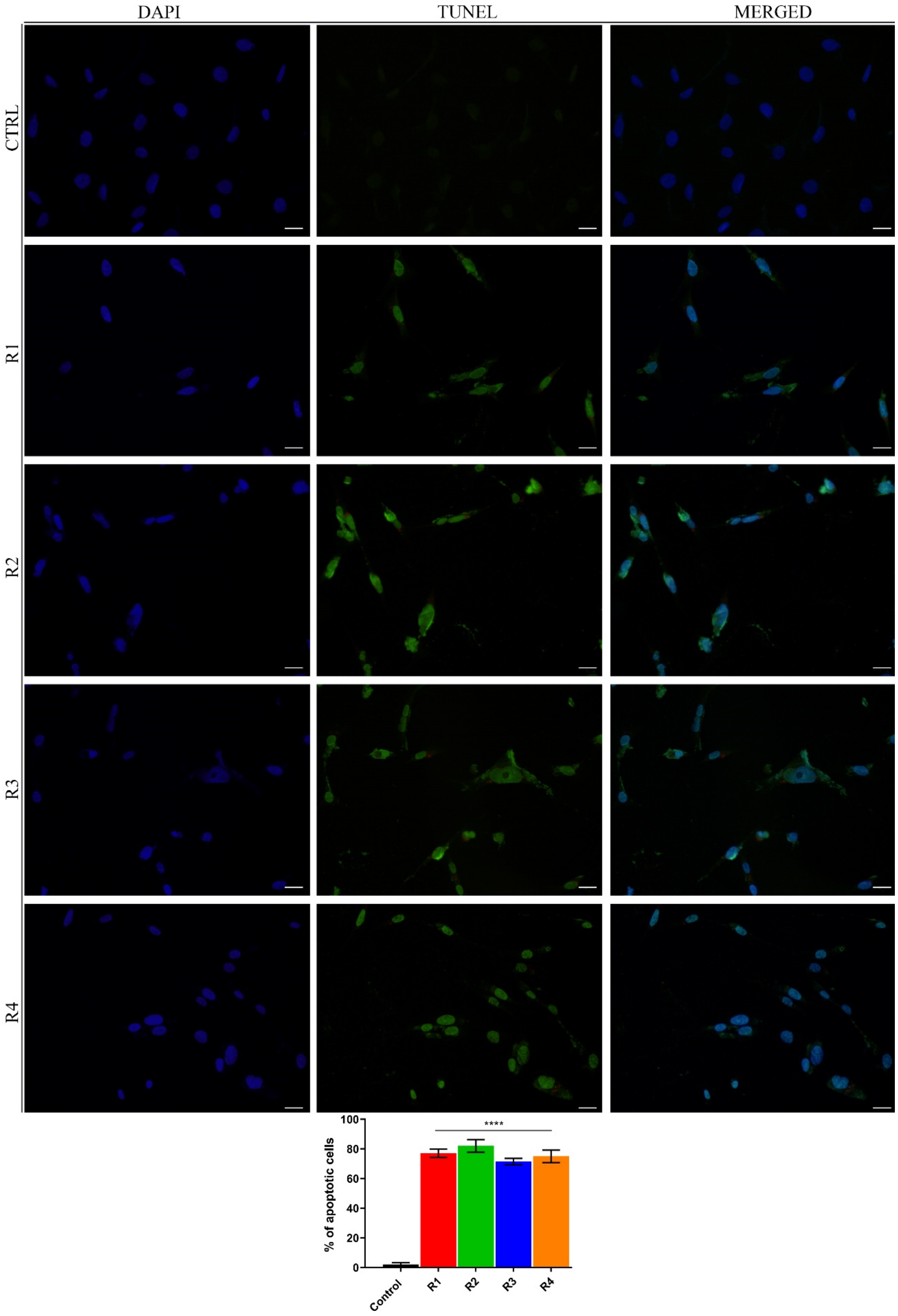

Figure S3. *S. rosmarinus* extracts induce cell apoptotic death. TdT-mediated dUTP nick-end-labeling (TUNEL) assay in MDA-MB-231 cells treated for 72 h with vehicle (Control) or rosemary extracts (R1-4). DAPI was used for DNA staining, scale bar: 50 μm. Histograms represent means ± S.D. of apoptotic vs control cells from three independent experiments performed in triplicate. *P* value were calculated against Control; **** P value < 0.0001.

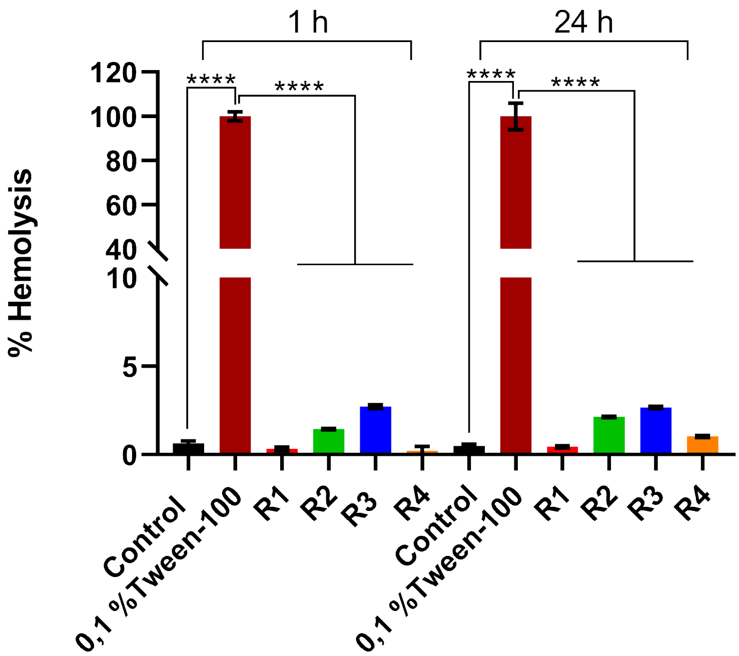

Figure S4. Rosemary extracts did not exert haemolytic effects. Haemolysis assay on RBCs treated with DMSO (Control), 0,1% Tween-100 or rosemary extracts (R1-R4), for 1h or 24 h. Histograms represent the relative percentage of hemolysis from three different experiments, each performed with triplicate samples. *P* value were calculated against 0,1% Tween-100; ****P value < 0.0001.

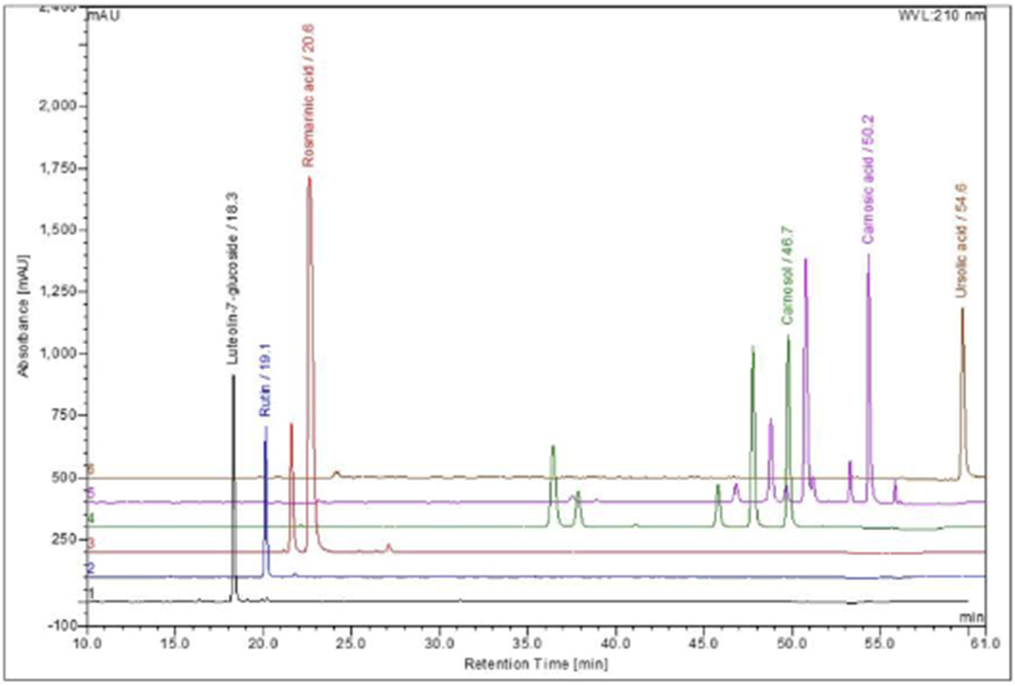

Figure S5. Standard compounds: luteolin-7-glucoside (0.26 g/L), rosmarinic acid (0.97 g/L), rutin (0.16 g/L), carnosol (0.51 g/L), carnosic acid (0.50 g/L), and ursolic acid (0.71 g/L).

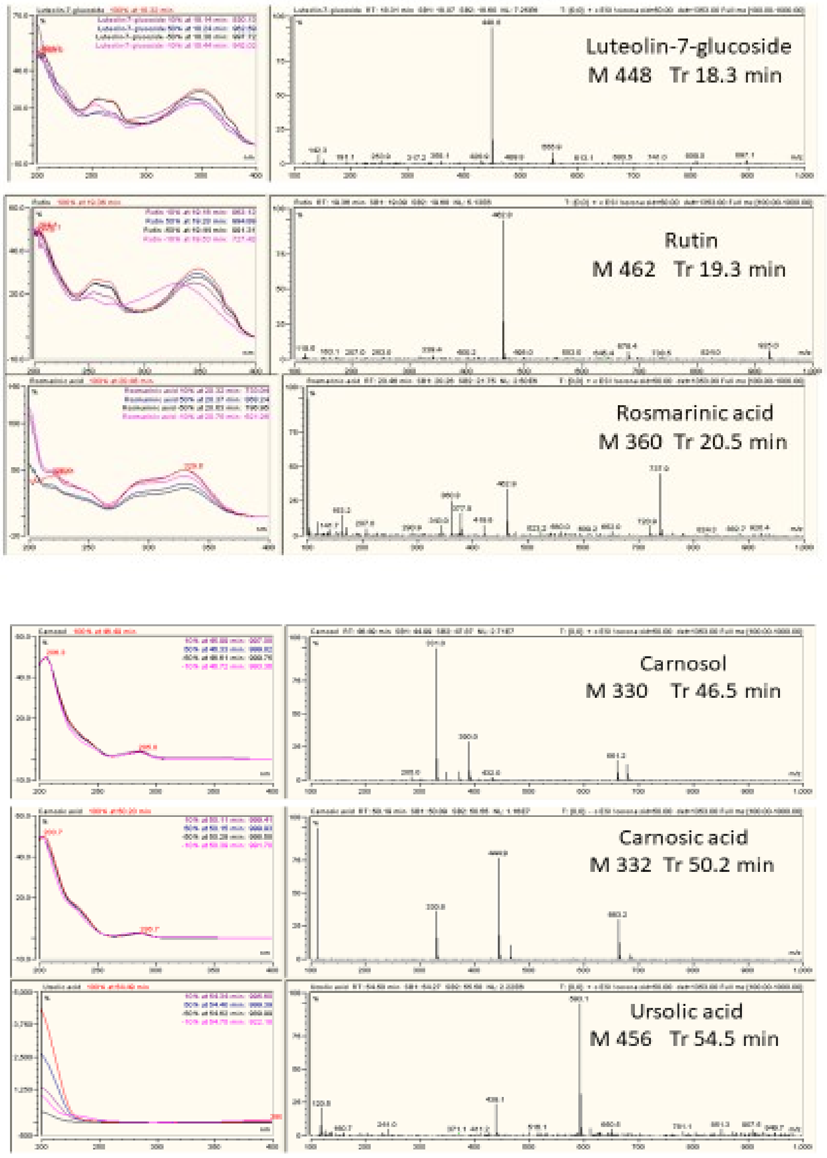

Figure S6. Standard compounds spectra.

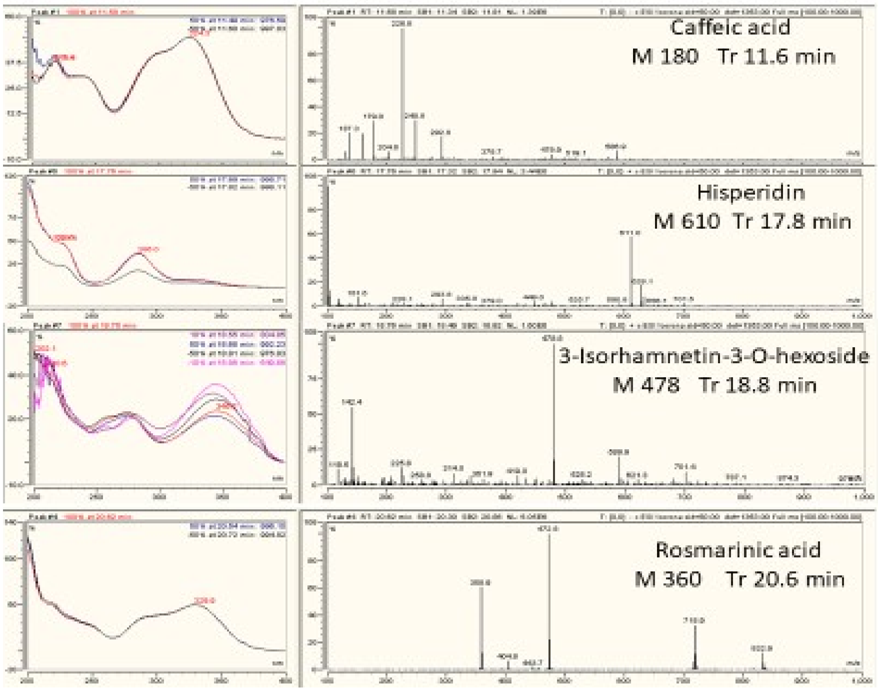

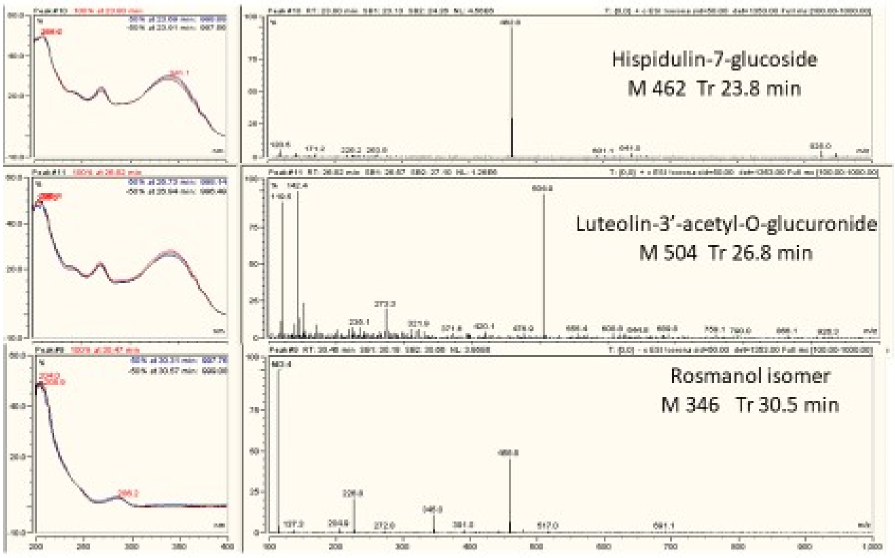

Figure S7. Sample compounds spectra.

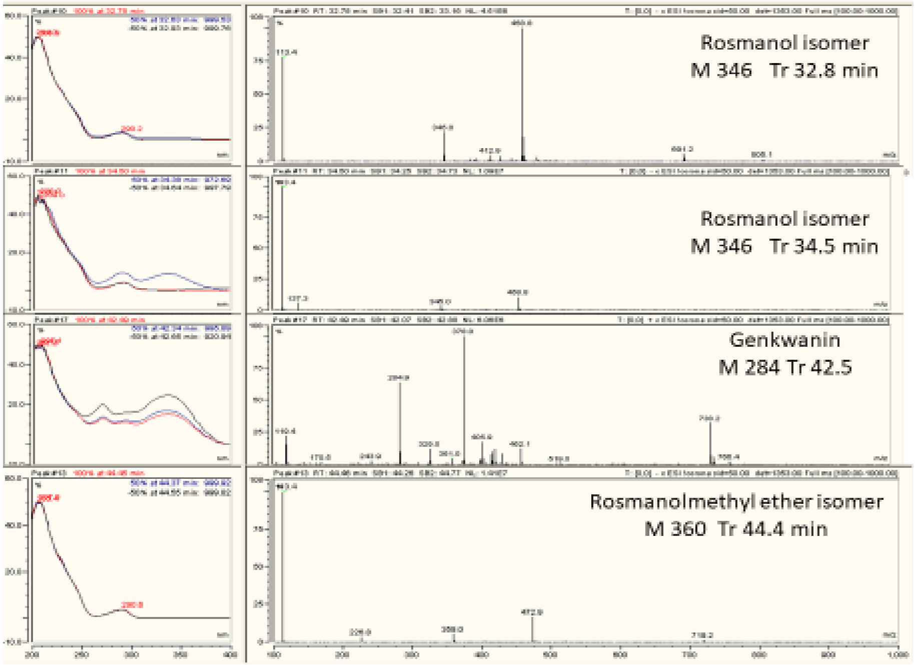

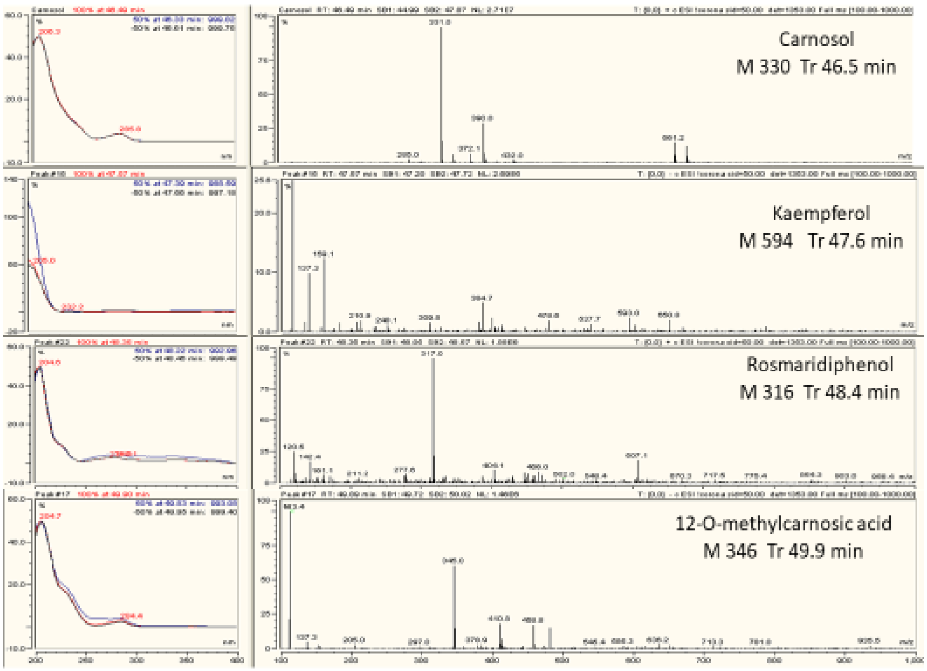

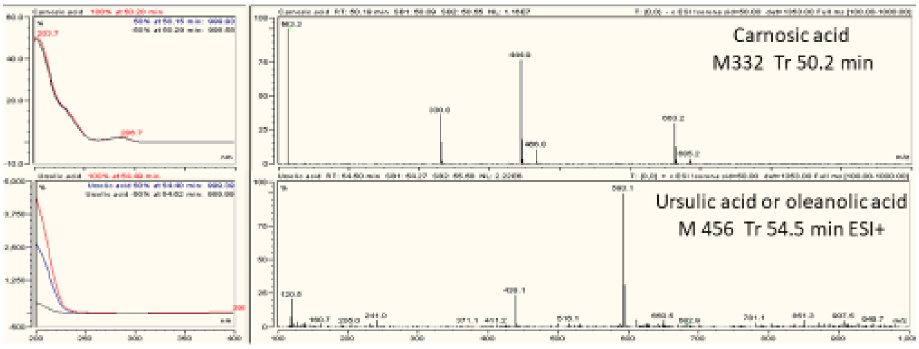
Carnosic acid
M332 Tr 50.2 min
Ursulic acid or oleanolic acid
M 456 Tr 54.5 min ESI+
